# Supplementary material for: Perceptions and practices of imaging personnel and physicians regarding the use of brain MRI for dementia diagnosis in Uganda
Source: PLoS One. 2025 Jan 17;20(1):e0305788. doi: 10.1371/journal.pone.0305788 (PMC11741567; doi:10.1371/journal.pone.0305788)
Supplement: Attachment — Submitted filename: Reviewer.docx [file pone.0305788.s002.docx]

Hello

Thank you for doing this research. The topic is interesting and adds to the literature since you point out that diagnosis of dementia remain challenging in LMIC and limited published study in MRI use in the same setting. However, you need to pay attention to the following comments to make your work worth-while.

1. **Abstract:**

- **Introduction:** The statement “In Uganda, MRI use for dementia diagnosis is infrequent” should be re-written as, “the use of MRI in diagnosis of dementia is infrequent in Uganda.” The statement “and subtle findings like mild regional atrophy are often overlooked, despite being crucial for imaging diagnosis” should be revised to make a clear meaning that connect well with the preceding statement. E.g., you could have said, “Even when it is used, subtle findings like mild regional atrophy are often overlooked, despite being crucial for imaging diagnosis.” Please revise and clarify the introduction in your own words.
- **Methods;** write the method clearly to include the study design, study setting, study participant, sampling method/sample size, data collection method, and data analysis. These should be short, clear and concise statements.
- **Results:** remove the number of participant from the result section. In qualitative research abstract, you could summarize your result by presenting the themes and the key message from the themes.

2. **Introduction;**

- You need to re-write and reorganize the introduction for clarity as well as improve the grammar. I have made a few but not all the suggestions to help you out.
- There is a lot of mix up of sentences; in paragraph 2 you are talking about situation in Uganda, then you brought definition and global perspective in paragraph three. Then in paragraph 4 you jumped to LMIC and back to Uganda in paragraph five. Is better to start with your brief opening statement, define, then global literatures/figures followed by LMIC and finally organize the local context/Ugandan literature in one paragraph
- Your definition of dementia should ideally come earlier than in paragraph 3.
- Where as in paragraph one you seem to imply that more than half of dementia is in LMIC, you invalidated this statement in paragraph 2
- There are statements in the last paragraph that needs referencing. Otherwise you are making unjustified claims.
- The last statement “the findings from the study will potentially help to identify ways of scaling up the use of brain MRI in the diagnosis of dementia especially in low resource settings in the wake of the increasing aging population.” is inappropriate in the introduction section and could appear as part of your conclusion or recommendation. It implies that you had pre-conceived result of your study prior to conducting it.
- Your introduction does not shade light into the practices of MRI use in dementia diagnosis globally, regionally or in the local context. You need to present what is known about your study topic (practice and perception) in global perspective or developed countries or LMIC. Otherwise this would imply that you are doing a ground breaking research.
- What is the current guideline or standard in dx of dementia whether in Uganda or in other settings
- What are the perceptions on the use of MRI in other setting?
- In summary, your introduction should make it clear as to where your research sits within the wider scholarly network, what is known about the topic, what the unknown is, and which gap in knowledge is it addressing.

3. **Methodology**

- Your methodology requires thorough revision. The sentences are poorly constructed with several grammatical errors and mix-up. I have a few questions and suggestions for clarifications and corrections.
- You have confused me with your study design. Please first state the study design, then in another paragraph you can write about the study setting and finally the data collection method. Otherwise you have mixed up the write up. Look at this example “This was an exploratory qualitative study. The study was conducted in……”
- Elaborate on your study setting; where in Kampala was the study conducted and what is the characteristic of your study setting.
- You mixed up your study settings and study participants. You are talking about the nature of MRI scanner in some facility as if those are part of the study participants.
- You used diverse category of participants. How did you sample the different groups of participant? How did you determine the sample size for each category of participants?
- Comment on your inclusion and exclusion criteria for each category of participants
- How where the study participants recruited?
- In which respective work place was the FGD conducted? You didn’t specify the work place of the study participant.
- Did you use a validated data collection tool? If not, how did ensure credibility of the data collected. Did you use the same data collection tool for all the different category of participant?
- How did you determine your saturation point and at what point did you achieve saturation for FGD and KIIs.
- You did only two FGDs involving two totally different category of participant. Please comment of data saturation for FGD and how you analyzed these two pieces of data.
- How did you analyze the data from the different categories of participants? Did you analyze them as aggregated data?
- Referencing in methodology is inappropriate. It is implied that your method is based on well-established methodology

4. **Results**

- You need to thoroughly improve the presentation of your results. Make your sentences clearer and correct use of grammar. You may need to re-visit your themes/codes and quotes again
- In your table of result you presented domains and themes yet in your data management and analysis you mentioned having generated codes, subthemes and themes.
- You had two objectives i.e., exploring perception and practice but your result does not to make it clearer for the reader how you addressed both objectives. I am interested in your results on the practice and perceptions as separate findings from the study.

5. The entire paper needs to be revised thoroughly for grammatical errors and clarity.
