# Supplementary material for: Perceptions and practices of imaging personnel and physicians regarding the use of brain MRI for dementia diagnosis in Uganda
Source: PLoS One. 2025 Jan 17;20(1):e0305788. doi: 10.1371/journal.pone.0305788 (PMC11741567; doi:10.1371/journal.pone.0305788)
Supplement: Attachment — Submitted filename: Response to reviewers.docx [file pone.0305788.s003.docx]

**Rita Nassanga**

Makerere University, College of Health Sciences

Department of Radiology and Radiotherapy

Date:19^th^. July.2024

**The Academic editor**

Plos-one

Dear Sir,

**RE: Response to the reviewer’s comments**

The issues raised by the editor have been addressed in the revised paper as summarized in the table below.

| **Serial #** | **Comment** | **Response** |
| --- | --- | --- |
|  | **ACADEMIC EDITOR:** The reviewers have given useful comments that will assist to further refine the paper. In addition to the reviewer comments, the authors need to strengthen the discussion by showing the relevance of this work to a global audience especially in LMICs where population demographics are changing and life expectancy is increasing yet use of MRI in the management of dementia has been less applied. Also, discuss the issue of limited training of the radiologists in use of MRI in dementia diagnosis. If they are not trained, this is likely to remain a challenge even after this study. In the methods section, the authors should show how rigor was ensured in the study since it was qualitative when it comes to issues of credibility, confirmability and dependability as well as transferability. How did the position of the researcher influence the research process? Is the researcher part of the people that were being studied? Issues of positionality have not been indicated in the methods. Lastly, please proof-read the entire paper to eliminate grammatical errors. | The discussion has been strengthened to show relevance of this work to a global audience.  The issues of rigor, credibility, confirmability and dependability as well as transferability have been addressed in the methods section. |
|  | **ABSTRACT** |  |
| 1. | **Abstract-Introduction:** The statement “In Uganda, MRI use for dementia diagnosis is infrequent” should be re-written as, “the use of MRI in diagnosis of dementia is infrequent in Uganda.” The statement “and subtle findings like mild regional atrophy are often overlooked, despite being crucial for imaging diagnosis” should be revised to make a clear meaning that connect well with the preceding statement. E.g., you could have said, “Even when it is used, subtle findings like mild regional atrophy are often overlooked, despite being crucial for imaging diagnosis.” Please revise and clarify the introduction in your own words. | Edited as suggested, in the introduction section of the abstract |
| 2. | **Abstract-Methods;** write the method clearly to include the study design, study setting, study participant, sampling method/sample size, data collection method, and data analysis. These should be short, clear and concise statements. | Edited as suggested, in the methods section of the abstract. |
| 3. | **Abstract-Results:** remove the number of participant from the result section.  In qualitative research abstract, you could summarize your result by presenting the themes and the key message from the themes. | Edited as suggested; the number of participants has been moved to the methods section of the abstract.  The themes, sub-themes and key messages have been stated in the results section of the abstract. |
|  | **INTRODUCTION** |  |
| 4. | You need to re-write and reorganize the introduction for clarity as well as improve the grammar. I have made a few but not all the suggestions to help you out. | The introduction has been re-written and organized as suggested. |
| 5. | There is a lot of mix up of sentences; in paragraph 2 you are talking about situation in Uganda, then you brought definition and global perspective in paragraph three. Then in paragraph 4 you jumped to LMIC and back to Uganda in paragraph five. Is better to start with your brief opening statement, define, then global literatures/figures followed by LMIC and finally organize the local context/Ugandan literature in one paragraph | The introduction has been re-arranged; the order is;  Definition of dementia, llobal literature, LMIC literature and lastly the Ugandan literature. |
| 6.. | Your definition of dementia should ideally come earlier than in paragraph 3. | The definition of dementia has been moved to paragraph one. |
| 7. | Where as in paragraph one you seem to imply that more than half of dementia is in LMIC, you invalidated this statement in paragraph 2 | This has been corrected. |
| 8. | There are statements in the last paragraph that needs referencing. Otherwise you are making unjustified claims. | Two references from studies done in Uganda have been added. |
| 9. | The last statement “the findings from the study will potentially help to identify ways of scaling up the use of brain MRI in the diagnosis of dementia especially in low resource settings in the wake of the increasing aging population.” is inappropriate in the introduction section and could appear as part of your conclusion or recommendation. It implies that you had pre-conceived result of your study prior to conducting it. | This statement has been moved to the conclusion section. |
| 10. | Your introduction does not shade light into the practices of MRI use in dementia diagnosis globally, regionally or in the local context. You need to present what is known about your study topic (practice and perception) in global perspective or developed countries or LMIC. Otherwise this would imply that you are doing a ground breaking research. | Previous studies on the Practices and perceptions on the use of brain MRI in the diagnosis of dementia, globally and locally, have been cited. |
| 11. | What is the current guideline or standard in dx of dementia whether in Uganda or in other settings | The standard of diagnosis in dementia in Uganda and other settings has been stated in paragraph four of the introduction. |
| 12. | What are the perceptions on the use of MRI in other setting? | Previous studies on the Practices and perceptions on the use of brain MRI in the diagnosis of dementia, globally and locally, have been cited. |
| 13. | In summary, your introduction should make it clear as to where your research sits within the wider scholarly network, what is known about the topic, what the unknown is, and which gap in knowledge is it addressing. | Further clarification has been provided in the introduction and all changes have been incorporated as suggested. |
|  | **METHODOLOGY** |  |
| 14. | Your methodology requires thorough revision. The sentences are poorly constructed with several grammatical errors and mix-up. I have a few questions and suggestions for clarifications and corrections. | Grammatical errors have been rectified.  The mix-up has been clarified. |
| 15. | You have confused me with your study design. Please first state the study design, then in another paragraph you can write about the study setting and finally the data collection method. Otherwise you have mixed up the write up. Look at this example “This was an exploratory qualitative study. The study was conducted in……” | The introductory part of the methodology has been clearly broken down into;    Study design  Study setting |
| 16. | Elaborate on your study setting; where in Kampala was the study conducted and what is the characteristic of your study setting. | The study took place at seven urban based health facilities in Kampala, Uganda, and each had a 1.5T MRI scanner. This has been clarified in the study setting |
| 17. | You mixed up your study settings and study participants. You are talking about the nature of MRI scanner in some facility as if those are part of the study participants. | The study setting has been re-written and the participants have been moved to *the participants and sampling section* of the methodology. The nature of the MRI scanner has been moved to the study setting. |
| 18. | You used diverse category of participants. How did you sample the different groups of participant? How did you determine the sample size for each category of participants? | All use MRI in several ways; clinicians refer, imaging techs carry out the MRIs whereas the Rads interpret these MRIs; we wanted to get a variety of responses from all stake holders /health professionals to maximum variation. |
| 19. | Comment on your inclusion and exclusion criteria for each category of participants | The inclusion and exclusion criteria for each category of participants has been stipulated in the methodology. |
| 20. | How where the study participants recruited? | This being a purely qualitative study, the participants were purposively selected on basis of their experience with brain MRI or diagnosis of dementia. The information has been included in the *the participants and sampling section* of the methodology. |
| 21. | In which respective work place was the FGD conducted? You didn’t specify the work place of the study participant. | The two FGDs were conducted in of the healthy facilities; the name of the specific facility has been left out due to ethical reasons of confidentiality. |
| 22. | Did you use a validated data collection tool? If not, how did ensure credibility of the data collected. Did you use the same data collection tool for all the different category of participant? | The questions for the FGDs and KIIs were informed by literature and first pre-tested with four experts to ensure their credibility. |
| 23. | How did you determine your saturation point and at what point did you achieve saturation for FGD and KIIs. | Saturation was achieved at the 10th interview however, one more was included to confirm saturation; this has been included in the revised paper. |
| 24. | You did only two FGDs involving two totally different category of participant. Please comment of data saturation for FGD and how you analyzed these two pieces of data. | These are two trainee groups involved in the assessment of dementia patients. However, carrying out only two FGDs could be a limitation of the study and has been included as part of the limitations in the discussion. |
| 25. | How did you analyze the data from the different categories of participants? Did you analyze them as aggregated data? | Yes the data was analysed as a whole because the FGD and KII questions were related. |
| 26. | Referencing in methodology is inappropriate. It is implied that your method is based on well-established methodology. | The steps that were followed were not for the researchers but were borrowed from previously documented literature. |
|  | **RESULTS** |  |
| 27. | In your table of results you presented domains and themes yet in your data management and analysis you mentioned having generated codes, sub-themes and themes. | A detailed table that shows the codes, themes, sub-themes and illustrative quotes has been included in the results section. |
| 28. | You need to thoroughly improve the presentation of your results. Make your sentences clearer and correct use of grammar. You may need to re-visit your themes/codes and quotes again | We have revisited the raw data and the quotes as well as the themes have been verified. We have also included a new detailed table showing the themes, sub-themes, codes and related quotes. |
| 29. | You had two objectives i.e., exploring perception and practice but your result does not to make it clearer for the reader how you addressed both objectives. I am interested in your results on the practice and perceptions as separate findings from the study. | The analysis followed an inductive approach where themes and sub-themes purely emerged from the data therefore, the perceptions and practices of the participants were indirectly expressed through the presented themes and sub-themes as seen in table 1. |
| 30. | The entire paper needs to be revised thoroughly for grammatical errors and clarity. |  |
| 31. | The manuscript seems to describe a technically sound piece of scientific research with data that supports the conclusions. However, there are several clarifications and corrections that needs to be done to make the manuscript clearer and reflect your objectives.  Whereas the author chose yes for the first question on data availability, no source of the data is provided instead the author assert that data cannot be shared publicly because of restrictions from Ethics Committee and that the data are available from the corresponding author on reasonable request.  There are several grammatical errors throughout the manuscript which can be dealt with at the rounds of review. | The whole paper has been revised and clarifications have been made.  Raw data will be available on individual request form the corresponding author due to IRB restrictions on data sharing.  The entire paper has been proof-read to correct the grammatical errors. |
| 32. | The use of "Practices" was not well defined in this manuscript. Its use in the results section looks more of MRI utilization or uptake as opposed to the actual practice. for example when you say Good practice, I would expect to see the appropriate application /use of MRI in the diagnosis of dementia; Poor practice may indicate some issues relatable to incompetency or in appropriate use of MRI or its abuse in the diagnosis of dementia. However what is indicated in Domain A of the results section are more of the influencers of MRI utilization or uptake; refer to the Domain 1,theme 3;Poor Practice (low patient numbers, cost of MRI) the attributable factors are more of the barriers mentioned in Domain C. | In the context of the study, practices referred to uptake or utilization of MRI. |
| 33. | There is need to have a consistent format in the quotations; i.e some quotes indicate participants age and experience well as other quotes only indicate experience. | The quotes have been edited for uniformity. |

Yours Sincerely,

**Dr. Rita Nassanga**

Corresponding author

Department of Radiology and Radiotherapy

College of Health Sciences, Makerere University.
