# Supplementary material for: Perceptions and practices of imaging personnel and physicians regarding the use of brain MRI for dementia diagnosis in Uganda
Source: PLoS One. 2025 Jan 17;20(1):e0305788. doi: 10.1371/journal.pone.0305788 (PMC11741567; doi:10.1371/journal.pone.0305788)
Supplement: Attachment — Submitted filename: Response to reviewers 2.docx [file pone.0305788.s005.docx]

**Rita Nassanga**

Makerere University, College of Health Sciences

Department of Radiology and Radiotherapy

Date: 02. Sept. 2024

**The Academic editor**

Plos-one

Dear Sir,

**RE: Response to the reviewer’s comments**

The issues raised by the editor have been addressed in the revised paper as summarized in the table below.

| **Serial #** | **Comment** | **Response** |
| --- | --- | --- |
|  | ACADEMIC EDITOR |  |
|  | The revised paper reads better though it still needs some refinement. In addition to the reviewer comments raised, please proof-read the paper to ensure that it flows well and correct the occasional grammatical errors. | The revised paper has been proofread after incorporating the reviewer comments. |
| 1. | One of the main problems in the paper is the poor organization of your write up, lack of clarity and a lot of grammatical errors. Work on the grammar thoroughly and improve clarity of your write up. You seem not to be paying careful attention to clear connection between sentences and paragraphs, grammar, and clarity. | The revised manuscript has been improved and proof read to correct the flow, grammar and clarity. |
|  | ABSTRACT |  |
| 2. | **Introduction:** The introduction should just be a snap shot and well aligned but not too verbose. If I delete most of your English, I end up with this short introduction. Think of how you can write a clear and concise introduction that does not obscure the main thing you want to present.  **“Introduction:** Diagnosing dementia remains challenging in low-income settings due to limited diagnostic options. Though brain MRI is crucial in dementia diagnosis, its use in Uganda remains infrequent.” | This has been revised as suggested. |
| **3.** | **Methods:** Change **“**and analyzed thematically using an inductive approach” to “and analyzed by inductive thematic analysis.” | This has been edited as suggested. |
| 4. | **Results:** You don’t need to list your subtheme here. After listing the themes, just present the key message from the result as the next statements. Please remove the subthemes and refines the remaining write up for clarity. | The subthemes have been removed as suggested. |
| 5. | Conclusions: your write up lacks proper coordination; re-write the conclusion and make the take home message very clear. How do you convince someone about adopting MRI use when your take home message is casting only doubt by presenting mainly draw-backs. You need to write and also provide positive side of your finding to convince the reader why MRI is worth adopting despite the barriers. | The conclusion was edited as advised. |
|  | MANUSCRIPT BODY |  |
| 6. | Your introduction is still so mixed up; you need to organize your argument into coherent write ups which connect well with each other. Please note that writing a compelling introduction/background is a sort of storytelling. There should be clear connection and flow of message from one paragraph to the next.  Your paragraph one could only focus on the definition and type/cause of dementia you stated. Please remove “it affects 7–8% of individuals older than 65 years old and 30% of individuals above 80 years old [8]” and find were to fit it under epidemiology/risk factors in paragraph 2 | The entire introduction has been revised as suggested to make it flow as suggested. It has also been proof-read to correct the grammar. |
| 7. | Then organize the paragraphs on epidemiology/prevalence and risk factor for dementia and connect the narrative well. I can’t find the clear flow in your paragraph two; you started globally, and then mention Africa, before jumping to LMIC then back to Africa/sub-Saharan Africa again. You could first present the global burden, then LMIC then Africa/sub-Saharan and finally the prevalence in Uganda. You can then bring in the drivers of dementia; you mentioned age as the major driver, but then connect it well with life expectancy in driving aging population. Then you can end the paragraph with the concerns related to the rising prevalence you tried to state. | The paragraphs have been reorganized as suggested. |
| 8. | Connect the third paragraph to the second using preposition such as despite, inspite of, although, etc. Example of a good start is “despite the rising prevalence of dementia, diagnosis is still very challenging…………………………………………………………………………….…………..” | This has been done. |
| 9. | The statement “model paper[14]In a previous study on perceptions on dementia diagnosis, it was reported that” doesn’t sit well. The sentence following it connects better to the preceding paragraph. You either delete that statement of find better way of putting it in that story you are trying to tell. | This has been deleted. |
| 10. | I am wondering how the lack of a diagnostic tool results in high prevalence of dementia in Uganda. Does that mean clinicians in Uganda are over diagnosing dementia? Please clarify on that statement and how that is possible, in way that a reader can get convinced. | This has been reworded and prevalence of dementia has been replaced with challenges in diagnosing dementia. |
| 11. | The last paragraph is good but you need to improve it. The sentences are too long; it also seems like you miss to put a full stop after the first sentence. | The sentences have been summarized and the paragraph has been improved. |
|  | METHODS |  |
| 12. | Study design; Remove the statement “conducted in health facilities with MRIs” because that appears in your study setting. | This has been done. |
| 13. | You don’t necessary to need to write inclusion and exclusion criteria as sub-headings. It should be seen in your description of the study participant. | The inclusion and exclusion criteria sub-headings have been removed. |
| 14. | What you have put as an exclusion criterion may not be correct for this study methodology. You exclude those who participated in the study. E.g. if for any reason you can’t analyze results from certain participant then you exclude them and give the reason for the exclusion. Similarly, you didn’t use convenience sampling. Therefore the absence of a participant can’t be an exclusion criterion for purposive sampling | This has been removed. |
| 15. | How long were the KII and FGDs? | Each KII lasted forty minutes whereas ech FGD lasted one hour. |
| 16. | Am still wondering how you used the same interview guide for technologist, radiologist psychiatrist, and Neurologist. These cadres interact with the brain MRI at different level. But if you were able to get what you set out to do then well and good. | The questions in the interview guide were formulated ton apply to all participant cadres because they were exploring their experience in general. |
|  | RESULTS |  |
| 17. | You may not need to put quotes in your table of result. It becomes a repetition as the same quotes appear down in the results below the table unless you are presenting the results in its entirety in a tabular form. | Not all quotes in the table are repeated in the description of the themes. The quotes in the narrative are just a small representation of the quotes in the table just to relate to each theme. The quotes in the table are meant to show the reader at a glance illustrative responses against each theme. In the last review comments, one of the reviewer srecommended to include some quotes in the table. |
| 18. | In your methodology, this is the participant categories you provided; the participants comprised three radiologists, six Radiology residents, eight Psychiatry residents, two psychiatrists, two neurologists, and four imaging technologists. However, the demographic categories presented in the result section are different. You now changed to “the sample included ten psychiatrists, two neurologists, three radiologists, and ten radiographers.” Such inconsistency is not good; you should be more alert in your writing to avoid such discrepancies and confusion. | This has been corrected in the results section to tally with the methodology. |
|  | DISCUSSION |  |
| 19. | Your discussions are also mixed up; the essence of discussion is to make the message from your finding clear and provide implication of your findings to practice.  In paragraph one you discussed limited skills in MRI interpretation. Paragraph two you discussed limited MRI access but in the last sentence you concluded with this statement “Similarly, a study conducted in USA highlighted challenges such as limited skills in interpreting MRI results, the high cost of neuroimaging and turnaround time for MRI results.” Stick to the main theme of the discussion in each paragraph, you have already discussed limited skills in the preceding paragraph. | This section has been revised to focus on skills only. |
| 20. | Looking at this part of your discussion, “A study by Boise on diagnosing Dementia, showed that some physicians only considered MRI if there was an unusually rapid progression of symptoms, a focus to the neurological symptoms when the patient was quite young. Whereas other physicians reported that one can't have a diagnosis of dementia without having had neuroimaging such as MRI [25] demonstrating a positive use of MRI in dementia diagnosis.” Put it in context of your findings rather than copying text. Try to re-write to bring the aspect of how participant in your study perceive prioritizing MRI. How they select patients for MRI investigation given the financial constraint. Bring clearly the key message of the participant. Do the same throughout your discussion, and if you have any key recommendation or gap based on your study, make them along. | This section has been revised to incorporate the changes as suggested. |
| 21. | Paragraph three, you are again discussing “insufficient training in brain MRI” which actually appeared in paragraph 1. Please merge the two discussions into one meaningful write up. | The two paragraphs on limited skills and access have been merged. |
| 22. | The statement; “participants identified claustrophobia and misconceptions about dementia as significant barriers to seeking diagnostic services, a finding corroborated by existing literature [30-33].” Please cite examples of the misconception from your finding before you continue the discussion. Whose misconceptions are being discussed here? Is it the public or practitioners’ misconception? | The misconceptions being referred to are of the patients. This has been clarified. |
| 23. | Give feasible recommendations that can improve practice and diagnosis of dementia in Uganda. Because based on your findings, the cost of MRI is the main prohibition of its use especially in the poor communities. I am wondering “what you mean by a phase rollout of MRI services” and how such a roll out will mitigate the financial constraints or increase patient’s capacity to afford brain MRI. | The recommendations have been revised. |
| 24. | Remember I asked you previously on how dementia is being diagnosed in Uganda (the current practice in diagnosis of dementia). True MRI is valuable and superior, but is only in Uganda Capital according to your literature. What do you recommend in the event that cost and limited access is involved and dementia patient are there in the communities not being diagnosed? | This statement has been included; In the beginning, the few available MRI centres need to be fully utilized by encouraging referral of dementia patients to these centres and coming up with a process of subsidizing costs for them. |
|  | **Conclusion** |  |
| 25. | Work on grammar and clarity. Provide concise, clear conclusions based on your findings. Remember you are making your case summary to convince someone on MRI adoption. How do you convince someone about adopting MRI use when your take home message is casting doubt by only presenting draw-back? | The conclusion has been revised to highlight the positive use of MRI in dementia despite the challenges. |

Yours Sincerely,

**Dr. Rita Nassanga**

Corresponding author

Department of Radiology and Radiotherapy

College of Health Sciences, Makerere University.
